# The Unresolved Paradox of Viable Microorganisms in Ancient Halite

**DOI:** 10.3390/life16101718

**Published:** 2026-10-14

**Authors:** Colby Renshaw, Yingguang Liu

**Affiliations:** College of Osteopathic Medicine, Liberty University, Lynchburg, VA 24515, USA; csrenshaw@liberty.edu

**Keywords:** halite, halophiles, microbial longevity, cellular dormancy, DNA damage, molecular repair, fluid inclusions

## Abstract

Reports of viable microorganisms from ancient halite create an unusual test of the biological limits of cellular survival. Halite deposits can be tens to hundreds of millions of years old, and increasingly rigorous studies have used chemical surface sterilization, petrographic selection of primary crystals, and direct sampling of primary fluid inclusions to reduce the likelihood of modern contamination. Nevertheless, the geological age of a crystal or inclusion does not by itself establish the age of a recovered organism. The central problem is that both major interpretations remain difficult. Modern contamination becomes less satisfactory when microorganisms are recovered from deeply buried, carefully sterilized primary material, yet uninterrupted survival for geological intervals is difficult to reconcile with spontaneous DNA and protein damage, ionizing radiation, finite energy supplies, and observed loss of microbial viability in other long-term environments. A further problem is molecular: several putatively ancient isolates or sequences are remarkably similar to modern organisms despite host-rock ages of tens to hundreds of millions of years. Proposed explanations include contamination, later recrystallization or groundwater infiltration, extremely low-activity maintenance states, and underestimation of genomic divergence by conserved markers such as 16S rRNA. No single explanation currently accounts for all reported observations and their heterogeneity in evidentiary quality. Ancient halite therefore provides a natural extreme-case model for a broader biomedical question: how long can a cell preserve sufficient molecular integrity for recovery when metabolism, repair, and replacement of damaged components approach their lower limits?

## 1. Introduction and Background

In October 2000, the reported revival of a Bacillaceae isolate from a brine inclusion of approximately 250-million-year-old Permian halite attracted considerable attention [1]. However, its genes were very similar to those of modern relatives [2]. The antiquity of the isolate was subsequently challenged, with modern environmental contamination proposed as an alternative explanation. Later efforts to isolate microorganisms from ancient halite found more archaea than bacteria (Table 1). Whole genomes of “ancient” microbes have been sequenced, and their phylogenetics and biochemical features have been analyzed [3,4,5]. Survival mechanisms of bacteria and archaea at extreme energy limits have been proposed and investigated [6,7,8,9,10]. Kinetics of biochemical processes that affect the lifespan of microorganisms have been studied at higher temperatures and extrapolated to environmental conditions [11,12]. Here we review a century of work on the identification and characterization of halophilic microorganisms in ancient halite, the properties of their genetic material, and proposed mechanisms of long-term survival.

For readers outside paleomicrobiology, the key distinction is simple but essential: the age of the salt deposit, the age of a fluid inclusion in the halite, and the age of a microorganism recovered from that inclusion are three separate claims. Geological and petrographic evidence can strongly support the first two without proving the third. The paradox arises because later studies increasingly reduced obvious routes of contamination while experimental biology simultaneously identified severe limits on how long an unrepaired or minimally active cell should retain a recoverable genome, proteome, and energy supply. This review evaluates both sides of that problem rather than assuming either ancient survival or modern contamination at the outset.

## 2. Why Ancient Halite Is Unusually Resistant to Contamination

Halite formations are widespread in the continental crust and can be massive in size. For example, the Salado formation from which the 250-million-year *Virgibacillus* was isolated covers 150,000 km^2^ with a thickness of 300–600 m. The ionic NaCl crystals are so compact that halite formations can be used to store radioactive waste and hydrogen gas [31,32,33]. The interior of intact halite beds can be strongly isolated from surface-water contamination. Even though halite may recrystallize under pressure, structural disturbances are localized, self-healing [34], and can be easily identified [35].

Microbes reside in fluid inclusions of halite crystals [36]. Fluid inclusions are small droplets of fluid, often less than 20 µm, that become trapped within a crystal either during the initial formation of that crystal or from a recrystallization event that occurred due to microfractures at a later time period [37]. Fluid inclusions formed during the initial growth of the crystal are termed primary inclusions, while those that occurred due to microfractures and recrystallizations at a later period are termed secondary inclusions.

The chemical composition of fluid inclusions in ancient halite is consistent with that of ancient seawater. Using a model based on steady-state mixing of river water with mid-ocean ridge hydrothermal brine, Spencer and Hardie [38] calculated ion compositions in seawater throughout geological times. Ion concentration fluctuations in ancient seawater derived from this model matched the observed geochemical alternations in nonskeletal limestones and potash evaporites during the past 600 million years [39]. Subsequently, Timofeeff et al. [40] analyzed the ion chemistry of primary fluid inclusions in halite crystals from multiple evaporite deposits, all from the Cretaceous period, and found that early Cretaceous halite inclusions had lower Mg^2+^/Ca^2+^ ratios than those in late Cretaceous halite, supporting the Spencer–Hardie model and the primary, syn-depositional origin of the inclusions, although inclusion age alone does not establish the age of microorganisms detected within them. Other physicochemical analyses have constrained the geological age and history of halite and its inclusions from geological periods such as Cambrian, Ordovician, and Permian [41,42,43].

## 3. How Contamination Controls Improved over Time

### 3.1. Pioneer Studies

In 1960, Reiser and Tasch reported finding diplococci in Permian halite crystals of the Carey Salt Mine in Hutchinson, Kansas, and subsequently culturing a halophilic Gram-positive diplococcus from dissolved crystals [13,44]. The crystals were submerged in 70% alcohol for 15 s before being dissolved in hyperosmotic media. At about the same time, Dr. Heinz Dombrowski of Justus Liebig University in Giessen, Germany, isolated multiple species of bacteria from halite from mines and drill cores of the ancient Zechstein Sea deposits [14,15]. He took scrupulous care in his lab setup, procedures, and sample preparation to eliminate laboratory contamination. He selected large primary salt crystals with diameters above 6 cm which “came from perfectly undisturbed points in the middle of larger successions of rock salt”, suspended them with a wire, flamed all sides for a total of one minute, cut the wire, and dropped the crystal into liquid medium in a glass tube which was then sealed and incubated at 40 °C. The treatment was tested and found to be efficient with halite crystals dipped in *Pseudomonas aeruginosa*. The medium was saturated with NaCl as the halite dissolved. His success rate was about 50%. He phenotypically identified one species as *Bacillus circulans* and another as a new species which he named *Pseudomonas halocrenaea*. Dombrowski also isolated bacteria from Precambrian (650 Myr), Silurian, and Devonian halite from North American samples. Dombrowski’s claims were so controversial at the time that, according to Sir Fred Hoyle, he lost his tenured position at Justus Liebig University. De Ley et al. [45] thoroughly analyzed the phenotypes and DNA composition of Dombrowski’s new *Pseudomonas* and found it indistinguishable from *Pseudomonas aeruginosa*. However, Bibo et al. [16] validated Dombrowski’s work by isolating *Bacillus* and other bacteria from Zechstein halite.

### 3.2. Revival and Expansion

Endeavors to isolate live microbes from ancient halite deposites revivied in the 1990s. Norton et al. [17] collected samples from two different British mines, the Winsford salt mine in Cheshire, England and the Boulby potash mine in Cleveland, England. The Winsford salt mine had bedded deposits from the Triassic period from 195 to 225 million years ago, and samples such as brine and recrystallized salt sludges from pools, wall efflorescence, and newly exposed surface crystals from tunnel walls by blasting were collected. The Boulby potash mine was located in a Permian formation from 225 to 270 million years ago, and samples such as halite, potash, brine pools, and efflorescence were collected. The surface crystals were submerged in absolute ethanol for 4–5 h for decontamination and transportation. Isolates taken from cultures of these samples were chemotaxonomically characterized by their lipid profiles and found to be comparable to several representative haloarchaeal genera including *Halobacterium (Hbt.) salinarum*, *Hbt. saccharovorum*, *Haloarcula*, and *Halococcus (Hcc.)*. While these isolates were from a variety of sample sources and not specifically fluid trapped within halite crystals, the authors argued for their syn-sedimentary origin due to the lack of typical surface colonization by haloarchaea in North Europe and the rapid loss of viability of these isolates in media without at least 1.5 M NaCl.

In an Austrian salt mine near Bad Ischl, Denner et al. [18] isolated a strain of *Halococcus* from 245 to 280-million-year-old Permian rock salt. The halite samples had clay on the surface, but they were dipped in ethanol and flamed [19]. The authors named the species *Hcc. salifodinae* and the isolate BIpT. The isolate shared similar features with previously sampled *Halococci* in 16S rRNA sequences, polar lipid composition, and menaquinone content, but differed in pigmentation, whole-cell protein patterns, and the presence of unknown lipids, justifying its classification as a new species. The group also isolated a species whose total cell protein constitution and membrane ATPase were similar to *Hbt. saccharovorum*.

Radax et al. [20] obtained halite crystals from mines and drill cores of multiple locations of Alpine halite deposits, flame-sterilized them, dissolved them in sterile saline, and collected bacteria by filtration. Colonies similar to *Hcc. salifodinae* grew from the samples. Using the same flame-sterilization and dissolution techniques, the same group identified another species of haloarchaea, *Hcc. dombrowskii* (named in honor of Heinz Dombrowski), in a newly blasted tunnel of the Bad Ischl mine [21], and a new species of *Halobacterium*, *Hbt. noricense*, from a drill core in a salt mine in Altaussee, Austria [23].

With many of these early studies, it is imperative to note that some of these isolates came from the bulk dissolution of rock salt without detailed specified surface-sterilization procedures or reported contamination testing. Ethanol is ineffective in killing bacterial endospores. Flaming the surfaces with controlled temperature change on the surface of the halite crystal as described by Radax et al. [20] may not achieve sterilization. Therefore, the validity of these results and antiquity of the resulting microorganisms relied more on repeated recovery of similar halophilic taxa across distinct geographic deposits, although contamination by microorganisms common to hypersaline environments cannot be excluded in these early studies.

By the turn of the century, PCR analyses of prokaryotic DNA in ancient halite started to appear in the literature, but we shall keep this review focused on the culturing of live organisms and compare them with PCR findings when relevant.

### 3.3. Refining Extraction Techniques

As the amount of literature exploring the extraction and isolation of halophilic microorganisms progressed throughout the years, researchers refined their techniques to better defend the antiquity of these species. Rosenzweig et al. [35] investigated chemical sterilization techniques and developed a method of reliable surface sterilization by treating halite crystals with 10 M NaOH and 1 M HCl, 5 min each. In addition, there was an emphasis on the identification and isolation of only primary halite crystals (instead of recrystallized crystals) and aseptic extraction of primary fluid inclusions. Armed with knowledge and skills of surface sterilization, halite crystal selection, and microscopic fluid extraction, the group isolated the famous 2-9-3 clone from the Salado formation, a Permian structure found in New Mexico, USA, which is dated at around 250 million years old by invertebrate fossils and radiometric aging [1]. Strain 2-9-3 is a moderately halophilic bacterium with the 16S rRNA gene 99% homologous to the spore-forming bacterium *Virgibacillus marismortui* and 97.5% homologous to *Virgibacillus pantothenticus*. Out of 53 crystals that were obtained from the formation, only two contained inclusions from which bacteria were grown, indicating a rare survival occurrence.

Mormile et al. [22] obtained halite samples in a drill core from Death Valley, California, 8 m and 85 m in depth (9600 and 97,000 years old according to U-Th dating). They examined fluid inclusions with a microscope and found bacterium-like microparticles in the halite inclusions of both depths. Subsequently, they used a microdrill and a micropipette to aseptically extract microparticle-containing fluid directly from primary inclusions of primary halite crystals. A live organism was isolated from 85 m deep halite but not from 8 m deep halite. The organism was identified by its 16S rRNA as the archaeon *Hbt. salinarum*.

Vreeland et al. studied microorganisms in the Sergipe Basin in Brazil, which contained salts from the Aptian stage in the early Cretaceous period dated between 121 and 112 million years old [24]. Fifty-six primary crystals in total were obtained and tested for fluid inclusion microbial growth after NaOH-HCl treatment, resulting in six microbial isolates from four crystals. Three of the crystals each contained one microbial isolate while one crystal contained three isolates. All six isolates belonged to the Archaea domain, with one from the genus *Natronobacterium* and five from the genus *Halobacterium*. Archaeal taxa were determined by lipid analysis along with 16S rRNA sequencing.

Gramain et al. [46] found that NaOH-HCl treatment failed to completely kill *Hcc. saccharolyticus* inoculated on the surface of halite crystals, nor did the procedure eliminate halococcal 16S rRNA on the crystal. Subsequently, they developed a more rigorous method consisting of sequential treatment with 6% sodium hypochlorite, 10 M NaOH, 10.53 M HCl, and 70% ethanol, each for 20 min. The method killed *Hcc. saccharolyticus* on the surface while sparing *Hbt. salinarum* entombed in inclusions. The group analyzed halophiles in a drill core obtained from the Salar Grande basin of Northern Chile dated to the Pliocene (1.8–5.3 million years old). While the top, recrystallized halite (not surface-sterilized due to small sizes) harbored diverse haloarchaea, the authors only isolated *Hbt. noricense* from surface-sterilized halite crystals deep in the basin and only found *Hbt. salinarum*-like 16S rRNA sequences in them. They tested the endurance of *Halobacterium* species in halite inclusions and found that *Hbt. noricense* recovered faster than *Hbt. salinarum* after 26 months of entombment in nutrient-free inclusions. *Halobacterium* species survived better than two other common halophiles, *Haloquadratum walsbyi* and *Salinibacter ruber*. The prominence of *Halobacterium* in deeper halite and the endurance of *Halobacterium* species in halite inclusions are consistent with the previous findings of Park et al., who analyzed ancient prokaryotic DNA in ancient salt crystals with primers for the 16S rRNA gene and found Halobacterium in Cretaceous and Silurian halite, while DNA of *Halorubrum* and *Haloarchula* was found in Oligocene crystals [26].

The concept that younger halite contains diverse haloarchaea is reinforced by a study by Schubert et al. [25] of a Pleistocene Death Valley salt core. They isolated *Halorubrum, Natronomonas*, and *Haloterrigena* from five of 881 crystals (surface-sterilized with 10 M NaOH) between 10,000 and 34,000 years old. Although the samples were primary crystals 13.0–17.8 m in depth, the microbiological profile was similar to the unsterilized, recrystallized surface halite of the Salar Grande. PCR analysis of 16S rRNA using the same drill core sample expanded the list of haloarchaea to include *Haloarcula*, *Halobiforma*, *Halosimplex*, *Halomicrobium*, and *Halonotius*, as well as abundant unclassified haloarchaeal DNA [47].

In addition to surface sterilization with concentrated NaOH and HCl, Jaakkola et al. [28] mechanically removed the outer shell of salt crystals by chiseling. They obtained eight haloarchaeal isolates from a rock salt drill core over 600 m in depth in the Jianghan Basin of Hubei Province, China. The sample was determined to be Eocene (38–41 million years old). Based on partial 16S rRNA sequences, two of the isolates were *Halobacterium* and the other six were *Halolamina*. Subsequently, the group obtained a 123-million-year-old Cretaceous drill core halite sample (2000 m in depth) from the same basin [4]. Using the same techniques, they isolated eight haloarchaeal colonies. Intensive genomic and phenotypic analyses demonstrated that the colonies belonged to a *Halobacterium* species whose 16S rRNA was 99% identical to that of *Hbt. noricense*, its closest phylogenetic relative. However, based on its low DNA–DNA re-association value (43%) with *Hbt. noricense*, the authors determined that the *Halobacterium* was a previously unidentified species and named it *Halobacterium hubeinese*. The two *Halobacterium* isolates that were previously obtained from the Eocene sample belonged to the same species (99.9% identity in 16S rRNA).

Meng et al. [29] further studied halophilic organisms in the Jianhan Basin using an Eocene halite sample (33.9–48.6 million years old) of a drill core that was rich in primary inclusions. To ensure surface sterilization, the authors cracked the crystals and treated them with concentrated NaOH and HCl, 10 min each. They dissolved the salt and isolated from it a bacterial species, an *Oceanobacillus* by 16S rRNA. It was the second member of the spore-forming Bacillaceae family (after the *Virgibacillus* revived in 2000) to be isolated from ancient halite.

Hoover and Pikuta [27] were the first group to isolate anaerobes (vibrio and rods) from Mississippian (350 Myr) halite as well as sylvite. They UV-irradiated crystals for 10 min after treating them in 70% alcohol before extracting the interior by fracturing them aseptically. They found more bacteria in sylvite than in halite.

### 3.4. Direct Microscopy

As early as 1935, Rippel [48] had dissolved newly blasted Permian halite crystals in water, stained the residues, and discovered bacterial cells, mostly rods. In 1963, Dombrowski [14] published a high-resolution photomicrograph of a rod-shaped bacterium “embedded in the crystalline structure of the salt” in thin sections. In 2003, Mormile et al. [22] were the first group to photograph bacteria in primary halite inclusions. Schubert et al. [49] provided a detailed morphological study documenting miniaturized cocci less than 1 µm in diameter in primary halite inclusions of 22 Kyr, 25 Kyr, and 34 Kyr halite crystals.

The most recent report on ancient halophiles was published in 2022, documenting microorganisms in the most ancient halite samples ever studied. Using halite crystals from the 830-million-year-old Browne Formation of central Australia, Schreder-Gomes et al. [50] examined bacteria-like structures in situ. A razor blade was used to cleave halite into 1–2 mm thick sections that were then scrupulously screened to only include primary fluid inclusions. Individual fluid inclusions were then visualized with both transmitted light and ultraviolet–visible light to detect optical appearance and fluorescent response. Through size, shape, and fluorescent response the authors were able to characterize the microorganisms as prokaryotes, eukaryotes, and organic compounds. Although this approach does not establish viability or the age of the observed structures, it provides in situ evidence for microorganism-like structures and organic material within primary fluid inclusions of Precambrian halite. Future improvements in sample preparation and optical technology may permit more detailed characterization of microorganisms or microbial remains in halite crystals.

A chronological list of live microbes isolated from ancient halite is provided in Table 1.

## 4. Why Geological-Age Survival of Endospores Is Biologically Difficult

The magnitude of the problem is easy to miss when geological ages are treated only as dates. A claim of survival for 100–250 million years requires a cell to preserve or repeatedly repair essential DNA, proteins, membranes, and other structures for intervals many orders of magnitude longer than direct laboratory observations of dormancy. Protection by salt can slow some reactions, but it cannot make spontaneous chemistry, natural radiation, or energy requirements disappear. The following evidence does not establish one universal expiration date for microbial life; instead, it shows why geological-age viability demands an additional mechanism or history of survival compared to what we know currently.

The following sections examine several biological constraints on the survival of microorganisms over geological timescales, beginning with bacterial endospores.

### 4.1. Biological Constraints on Endospore Longevity

An endospore is a dormant form produced mainly by members of Bacillaceae and Clostridia [51]. Its dehydrated core contains the bacterial genome, small acid-soluble proteins, and other molecular components protected by a multilayered coat [52,53]. Extremely long survival times have been proposed for dried bacteria and endospores [15,51]; however, indefinite persistence would require spontaneous molecular damage to remain negligible or repairable over time. Residual water permits slow hydrolytic reactions, while environmental radionuclides can cause direct and indirect damage. There is evidence that dose-rate dependence of ionizing radiation is minimal, meaning that comparable accumulated doses delivered at lower dose rates over longer periods can cause similar damage to higher doses over a shorter duration [54]. Temperature accelerates many damaging chemical reactions [55]; for example, dry storage of *Bacillus subtilis* var. *niger* spores at 37 °C caused substantial loss of viability over weeks [56], whereas higher temperatures (65–70 °C) can damage spores over hours [57]. The thermal history of individual halite deposits therefore matters when evaluating proposed survival intervals [14]. Because dormant spores lack the ongoing repair and replacement of biomolecules, accumulated lesions must remain compatible with successful germination and repair during revival [53]. The following sections examine radiation, DNA and protein degradation, and empirical changes in viable-spore abundance in sediments.

### 4.2. Irradiation by ^40^K Limits Spore Survival Time

In a direct response to the report of strain 2-9-3 from the approximately 250 Myr Salado Formation [1], Kminek et al. examined whether natural ionizing radiation would constrain the persistence of bacterial spores in halite fluid inclusions [58]. Their model treated ^40^K as the principal radionuclide in the brine inclusion and combined calculated dose accumulation with experimentally measured radiation-inactivation constants for spores, including the halophilic spore-former *Virgibacillus marismortui*. Assuming a 10 µL inclusion and an initial bacterial concentration of 4000 CFU/mL based on counts by Vreeland et al. [59], the calculated chance of finding a viable spore fell to about 1 in 25,000 after 17–34 Myr at 7 g/L potassium (average concentration in fluid inclusions) and 55–109 Myr at 2.2 g/L potassium (low potassium fluid), depending on the inactivation constant used. The authors therefore concluded that, in the absence of active repair during dormancy, ionizing radiation from ^40^K within the inclusion body would impose an upper survival limit of less than 109 Myr under the modeled halite conditions.

The calculated upper limits (<109 Myr) assume no active repair and a fixed initial cell density in a closed 10 µL inclusion. Real inclusions are often much smaller, potassium concentrations vary, and any residual metabolic activity (even intermittent) would change the outcome.

### 4.3. Degradation of DNA

DNA is a relatively unstable biomolecule and undergoes spontaneous base modification, hydrolysis of phosphodiester and N-glycosidic bonds, oxidation, and alkylation [60]. The N-glycosidic linkage between deoxyribose and purine bases is particularly labile, with depurination strongly dependent on temperature and chemical environment [61,62]. Dormant spores cannot continuously repair such damage; lesions can therefore accumulate until germination, when survival depends on the extent of protection and on successful repair during revival [63]. Johnson et al. [64] examined bacterial persistence in permafrost samples spanning up to approximately one million years by combining long-fragment PCR, uracil-N-glycosylase treatment, and low-temperature respiration measurements. Four-kilobase bacterial DNA was recovered from 400 to 600 kyr samples but not from samples dated to 740 kyr or approximately 1 Myr. Respiration was also detected in samples younger than 600 kyr but not in the 740 kyr sample. In addition, they found evidence of significant cytosine deamination in low-GC Gram-positive spore-forming bacteria. Notably, the oldest amplifiable DNA was dominated by non-spore-forming Actinobacteria rather than recognized endospore-formers. These observations support bacterial survival to roughly half a million years under the studied permafrost conditions, but they do not establish a universal maximum survival time for bacteria or spores. The authors instead argued that low-level metabolism and DNA repair can be more effective than strict dormancy for very long persistence.

DNA degradation rates in ancient halophiles remain uncertain. Processes such as depurination are sensitive to ionic strength and increasing NaCl concentrations have been shown to suppress depurination in aqueous conditions tested [65,66]. However, these measurements have not reproduced the ionic strength, low water activity, and DNA–protein interactions relevant to microbial survival in ancient halite inclusions.

### 4.4. Degradation of Proteins

Spontaneous degradation of proteins has also been extensively studied. From measurements of free and peptide-bound amino acids in deep-sea drill cores, Bada and Man [67] estimated a peptide-bond hydrolysis half-life of approximately 1–2 Myr in calcareous sediments, while noting that hydrolysis may be considerably slower in non-carbonate sediments. This is a molecular-diagenesis timescale rather than a direct measurement of microbial viability. Amino-acid racemization provides another cumulative measure of molecular alteration, and aspartate is among the relatively rapidly racemizing residues [68]. An experimental study related aspartate racemization to loss of viability in *Geobacillus stearothermophilus* spores and extrapolated measured high-temperature kinetics (50–127 °C) to environmentally relevant temperatures [12]. For Aarhus Bay conditions of 0–15 °C, the model predicted spore half-lives of 40–421 years; for Lake Constance sediment at 4 °C, the predicted half-life was about 220 years. These estimates depend on extrapolation and on environmental variables such as temperature, pH, ionic strength, and matrix effects, but they are broadly consistent with viability-based estimates from cold sediments.

Data on high-temperature inactivation of endospores provide an instructive warning about long extrapolations. Nicholson [11] compiled thermal-inactivation data for spores of several *Bacillus* species and extrapolated them to the estimated 25–40 °C temperature range of the Salado Formation. For a bacterial group containing moderately thermophilic *B. stearothermophilus* strains, the extrapolated D-values ranged from approximately 1.9 × 10^9^ to 1.9 × 10^12^ years. Nicholson explicitly noted that the upper values were physically nonsensical and emphasized that no direct thermal-inactivation data were then available for spores in brine. The exercise therefore demonstrates that a mathematically good fit over an experimental temperature range does not guarantee a biologically meaningful extrapolation across geological timescales.

### 4.5. Observed Decline of Spore Counts in Deeper Sediments

Several sediment studies have reported approximately exponential declines in the abundance of culturable or viable endospores with burial age. De Rezende et al. [69] studied thermophilic sulfate-reducing endospores in Aarhus Bay and obtained an estimated culturability half-life of approximately 330 years. A related study of thermophilic fermentative endospores at the same site reported an exponential decrease with depth and an estimated half-life of 350 years [70]. Rothfuss et al. [71] found that viable aerobic and anaerobic heterotrophic spores in Lake Constance sediments decreased with depth and fell below detection in approximately 8900-year-old sediment. Their reported death-rate constants of 0.0013–0.0025 year^−1^ correspond to first-order half-lives of approximately 277–533 years. These values describe population-level loss of viability or culturability in sediment under specific depositional and thermal histories and should not be interpreted as universal intrinsic half-lives of individual spores. Wörmer et al. [72] used dipicolinic acid (DPA) as a culture-independent proxy for endospore abundance across a global marine-sediment dataset. A segmented regression showed a breakpoint near 14.2 kyr: endospore concentration declined by more than two orders of magnitude over the younger interval, whereas across the approximately 20 Myr represented by the older segment the DPA-derived concentration was only halved. This latter value is therefore an abundance trend across heterogeneous sediments, not a 20 Myr measured viability half-life. Although DPA is diagnostic of endospores and the authors argued against a major contribution from fossil DPA, DPA quantification itself is not a direct viability assay.

Viable bacterial concentrations in brines are about 4000 CFU/mL as reported [59]. Laboratory experiments indicate that only a minor fraction of haloarchaea survive during incorporation into halite. Kixmüller and Greie [73] observed that approximately 10% of wild-type *Hbt. salinarum* cells remained viable following entombment in halite, a value they considered consistent with earlier observations by Fendrihan et al. [74] and Kottemann et al. [75]. As an illustrative calculation, suppose that 10% of those cells remain represented as viable spores after entrapment, giving 400 spores/mL of inclusion brine. If fluid inclusions occupy 2% of halite volume [76] and halite density is 2.16 g/cm^3^, one metric ton of halite contains approximately 9.26 L of inclusion fluid and therefore about 3.7 × 10^6^ spores under these assumptions. Applying the longest half-life of spore viability in lake-floor sediments as estimated above, 533 years, a simple first-order decay model would reduce the expected number below one after approximately 11,600 years. This calculation is a population-level extrapolation, not a universal survival limit.

## 5. The Energy Problem for Live Cells in a Closed Fluid Inclusion

While the lifespan of a dormant endospore is limited by accumulation of unrepaired molecular damage, a cell with any level of metabolism faces a starvation problem when entombed in a small drop of saline. Recent developments in microbiology revealed a couple of survival mechanisms for starved microorganisms, whether they form spores or not.

### 5.1. Long-Term Stationary Phase (LTSP)

The bacterial growth curve consists of five phases: lag phase, log phase, stationary phase, death phase, and long-term stationary phase [7,77]. The long-term stationary phase (LTSP) commences when most of the microbial population dies and releases nutrients into the environment, initiating nutrient recycling by cannibalism [78]. *Escherichia coli* (*E. coli*) was found to survive in broth cultures for at least 5 years [77]. During LTSP, microorganisms adapt and gain the ability to survive under various environmental stress. When deeply starved, most *Bacillus subtilis* cells sporulate, but others assume a slow-growing coccoid shape with their doubling time prolonged from ~40 min to ~4 days [79]. The DNA-binding Protein from Starved cells (Dps) becomes the most represented protein in *E. coli* cells and protects the cell and its DNA from ensuing oxidative stress [80]. Homologues of Dps have been found in other bacteria and archaea [81,82,83].

To examine the persistence of living cells under extreme energy limitation, Shoemaker et al. [84] followed 21 heterotrophic bacterial taxa in the long-term stationary phase at 25 °C in darkness and modeled mortality trajectories. The resulting estimates of population time to extinction spanned roughly 1–100,000 years, with large differences among taxa and especially long persistence among spore-forming *Bacillus*. These are model-based population-extinction times from laboratory starvation experiments rather than direct measurements of continuous survival over those intervals. Gray et al. [79] further showed that long-term *Bacillus subtilis* populations can persist without repeated wholesale cycles of sporulation and germination, underscoring that LTSP survival is a population-dynamic process distinct from simple endospore dormancy.

It is important to note the thermodynamic cost of cannibalism in LTSP. Schink et al. [78] found that it took 5.5 dead *E. coli* cells to make one new living cell. When *E. coli* enters LTSP, numerous mutations that increase the efficiency of energy use (growth advantage in stationary phase, GASP) are selected, most notably those inactivating the *rpoS* gene that codes the general stress response sigma factor. By partially inactivating *rpoS*, these mutants demonstrate enhanced catabolism of amino acids as alternative carbon sources. Paradoxically, *ΔrpoS* mutant cells increase the death rate by 1.8-fold and increase the number of dead cells to build a new cell to 10, essentially creating a bacterial population that extinguishes quicker [78,85]. Because of the improvisational nature of natural selection, GASP may only mean transient advantage instead of long-term survival. Indeed, carbon-efficient *E. coli* cells out of LTSP demonstrated reduced survival time in the presence of a producer organism, *Chlamydomonas reinhardtii* [86].

Assuming a bacterial concentration of 4000 CFU/mL [59], the number of organisms originally trapped in primary inclusions should be small. If we assume a generous number of 100 organisms in a fluid inclusion, and it takes five dead cells to grow a live cell, it only takes three cycles to reduce the number of live cells to less than one. Thus, cannibalistic recycling alone appears insufficient to sustain a small, closed halophile population for millions of years in a brine inclusion.

Cannibalistic LTSP has not been demonstrated in haloarchaea. However, nutrient recycling may play a role in their survival in halite inclusions. The *Halobacterium* genus has the unique ability of arginine fermentation, and arginine-rich media has been shown to enrich *Halobacterium* from saltern brine samples in anaerobic cultures [87]. This is consistent with *Halobacterium* predominance in more ancient halite. Even though arginine constitutes only a small percentage of the haloarchaeal biomass and is therefore inefficient in supporting cell division, arginine recycling may play an important role in starvation survival at lower rates of metabolism (see below). Additional metabolic pathways may provide other possible explanations. For example, genomic analyses indicate that haloarchaea in the Antarctic Deep Lake have the capacity to deaminate branched-chain amino acids, generating 2-oxoacids that can be further catabolized to citric acid cycle intermediates [88]. Whether such pathways can contribute to energy production under the anoxic and nutrient-limited conditions of ancient fluid inclusions remains unknown.

### 5.2. Haloarchaeal Microspheres

Although archaea do not form endospores, they are known to endure long-term starvation, which may have to do with nutrients stored in their polyploid genome [28]. Haloarchaea were revived from Dead Sea brine samples enriched with 1% peptone after 56 years of laboratory storage [89]. Many plants and microbes, including halophilic bacteria such as *Vibrio*, *Aeromonas*, *Pseudomonas*, and *Alcaligenes*, undergo miniaturization in adverse environments [90,91,92]. Archaeal cells also undergo similar morphological changes under severe starvation. In a 56-day starvation experiment, populations of *Hbt. salinarum* and a Death Valley *Haloterrigena* isolate (DV582A-1) showed a progressive shift from predominantly long rods toward smaller, rounded or coccoid forms [93]. Rods were still observed in Saline Valley halite inclusions up to 26 years old, whereas virtually all prokaryotic forms in 31 kyr old Death Valley core samples were coccoid [49]. Schubert et al. were able to revive haloarchaeal spheres from ancient Death Valley samples [25]. Unlike endospores, microspheres were observed to form ‘nearly instantaneously’ when rod-shaped *Halobacterium* cells were exposed to sufficiently low external water activity, with each rod producing three to four approximately 0.4 µm spheres [94]. Haloarchaeal polyploidy could potentially allow multiple genome-containing spheres to form from a single rod without requiring de novo DNA replication. *Halobacterium* microspheres remained viable in laboratory conditions for at least 6 years without organic nutrients and can reverse to rod shape when water activity increases. While the rod and spherical forms of the *Halobacterium* genus displayed many of the same biochemical properties, there was a markedly large difference in ATP content, with the microspheres containing approximately 50-fold less ATP than the rods. Taken together, these observations are consistent with microsphere formation being a reversible, stress-induced, dormancy-like morphological and physiological response. Compared with the 240-fold reduction in ATP content in bacterial endospores [95], haloarchaeal microspheres may retain some metabolic activity, although ATP content alone does not establish the rate or physiological purpose of that activity. Besides their polyploid DNA, haloarchaeal microspheres may not possess more mechanisms of survival than non-spore-forming halophilic bacteria. *Pseudomonas aeruginosa*, for example, was found to survive for long periods of time (at least 14 years according to Elabed et al.) in the inclusions of lab-grown halite crystals [96,97], in partial vindication of Heinz Dombrowski.

Haloarchaeal microspheres can coexist with cells or remains of the alga *Dunaliella* in brine inclusions [98,99]. *Dunaliella* can accumulate very high intracellular glycerol concentrations, and glycerol has been proposed as an important carbon and energy source for co-trapped haloarchaea [6]. A published energy-budget calculation based on a 10 µm *Dunaliella* cell containing 5.5 M glycerol and a miniaturized 10-fg-C prokaryotic cell estimated that this carbon reserve could support repair metabolism for approximately 12 Myr under a maintenance metabolic rate of 10^−7^ h^−1^ [98]. The assumed rate was derived from the community-level metabolic rates compiled by Price and Sowers [100], in which rates on the order of 10^−7^ h^−1^ fall within the range attributed to maintenance metabolism at subfreezing temperatures. Because biochemical degradation rates generally increase exponentially with temperature, the applicability of this subfreezing maintenance rate to subsurface salt beds under different geothermal conditions is uncertain.

This 10^−7^ h^−1^ rate corresponds to only 9.1 × 10^−21^ W per cell. Subsequently, Kempes et al. [101] adopted 3.17 × 10^−20^ W cell^−1^ as a lower-bound estimate of the basal power requirement, based on the minimum cell-specific energy turnover inferred by Lever et al. [91] for sulfate-reducing communities in energy-limited subsurface sediments. Other directly inferred maintenance energy requirements were orders of magnitude higher [102,103].

Thus, the 12 Myr estimate represents an assumption-sensitive upper bound of survival time in a small inclusion body with a coexisting algal cell. In addition, the usable energy yield depends heavily on redox conditions and metabolic pathways within the inclusion. There is no evidence that haloarchaea can metabolize glycerol anaerobically [104], while halophilic bacteria such as *Virgibacillus* may ferment glycerol, producing acid [105]. Fermentation only extracts a small fraction of the usable energy of glycerol, and the resulting accumulation of acidic waste products within a highly confined, poorly buffered microenvironment could lower the internal pH and further constrain the usable survival window long before the theoretical 12 Myr energetic limit could ever be reached.

Table 2 summarizes quantitative estimates relevant to microbial survival time over geological timescales.

## 6. The Molecular-Age Paradox: Why Do Ancient Isolates Look Modern?

Over sufficiently long evolutionary intervals, independently evolving microbial lineages are expected to accumulate sequence differences, although substitution rates vary substantially among taxa, genes, and environments [106]. While halophiles entombed in fluid inclusions may remain dormant, their free-living relatives continue to reproduce and evolve. Thus, if recovered microorganisms are truly contemporary with the halite, some genetic divergence from their modern relatives would be expected, even if little or no replication-associated evolution occurred in the entombed lineage.

The *Bacillus* strain isolated from 250 Myr Permian salt, strain 2-9-3, was 99% identical in 16S rRNA to modern *Virgibacillus marismortui* from the Dead Sea [1]. To further compare the DNA of 2-9-3 and *Virgibacillus marismortui*, Maughan et al. [107] partially sequenced two protein-coding genes, *recA* and *splB*, in both strains. The *recA* gene is conserved throughout the bacterial domain, while the latter is unique to Gram-positive spore-formers. The two organisms demonstrated two synonymous substitutions among the 404 nucleotides of the *recA* gene and one synonymous mutation among the 619 nucleotides of the *splB* gene. In other words, they share identical protein sequences. The RecA and SplB proteins of other extant *Bacillus* species are all different from those of 2-9-3 to various degrees. The extremely small number of differences in these two protein-coding genes therefore remains difficult to reconcile with prolonged genetic isolation over approximately 250 million years. Using the synonymous-site density of the closely related *Virgibacillus salexigens* DSM 11483, we estimate the synonymous substitution rate (*r_s_*) for *recA* and *splB* to be 8.9 × 10^−11^ and 3.1 × 10^−11^ per site per year, respectively. As a comparison, the *r_s_* of *E. coli* and *Salmonella enterica* based on homologous protein-coding genes is 4.5 × 10^−9^ per site per year [108].

Even though archaea reproduce slower than bacteria under optimum laboratory conditions, their generation times in the wild are primarily determined by environmental factors such as nutrient availability, temperature, and bacteriophages [109,110,111]. Most bacteria in the wild are in the long-term stationary phase [112,113,114]. Based on absorption of ^3^H-labeled thymidine, the growth rates of halophilic archaea were found to be comparable to those of bacteria in saltern ponds of various salinity levels [115,116]. In the higher temperatures of hypersaline environments, halophilic archaea can achieve faster growth than many bacteria, with doubling times between 1.5 and 3 h [110].

On the other hand, observed per-generation genome-wide mutation rates of halophilic archaea are also comparable to those of mesophilic bacteria [117,118]. Even though the polyploidy of archaea may facilitate recombinational DNA repair, polyploidy also relaxes natural selection and has been found to facilitate accumulation of deleterious mutations in plants [119]. Genomic streamlining favors deletion in multicopy genes [120,121]. *Halobacterium salinarum* and *Haloferax volcanii* both feature high rates of indel mutations with a bias toward deletions [118,122]. These observations do not support the assumption that haloarchaea as a group possess slower molecular-evolution rates than bacteria.

We identified and analyzed three published genomes of putative ancient haloarchaea, *Hbt. noricense* from Altaussee, Austria, *Hbt. hubeiense* JI20-1 from Hubei, China, and *Halobacterium* sp. Strain NMX12-1 from New Mexico, USA. The ages of the halite in the three places are 250 Myr, 123 Myr, and 250 Myr, respectively. Interestingly, a modern strain of *Hbt. hubeiense* was isolated from a solar saltern in South Korea and its genome was subsequently sequenced [123]. We compared the 16S rRNA and whole genomes of the above-mentioned archaeal strains (Table 3). We also compared their *radA* gene, a homolog of the bacterial *recA* (Table 4). One surprising finding is the high homology between *Hbt. hubeiense* JI20-1 and the American *Hbt. sp.* NMX12-1. Their 16S rRNA genes are 100% identical and the OrthoANI calculated using EZBioCloud, 96.86%, exceeds the 95% threshold for species definition [124], notwithstanding a temporal separation of 127 Myr and a geographic separation by the Pacific Ocean. On the other hand, the modern Korean *Hbt. hubeiense* and the JI20-1-type strain, separated by a comparable 123 Myr but only about 800 miles from each other, show an OrthoANI of only 88.75%. The OrthoANI values between JI20-1 or *Hbt. noricense* and seven other modern *Halobacterium* species range between 78.3% and 82.6%, while the OrthoANI values between the modern *Halobacterium* species lie between 78.0% and 82.6% ([125] and authors’ own calculation to include the Korean strain of *Hbt. hubeiense*). The fact that homologies among all three ancient genomes are higher than those among modern genomes is compatible with diversifying evolution through history, but the very high identity between the ancient Chinese isolate and the more ancient American isolate is difficult to reconcile with their reported geological ages and geographic separation under a simple model of continuous independent evolution. For comparison, *E. coli* and *Salmonella enterica* diverged from each other within a similar time range of 100-160 Myr [108]. The OrthoANI between *E. coli* DSM30083 and *Salmonella* is 80.6%.

The apparent substitution rates inferred from the *radA* gene of ancient archaea seem to be higher than those inferred from the *recA* gene of 2-9-3, but the apparent synonymous substitution rates between *Hbt. sp*. NMX12-1 or *Hbt. noricense* and later relatives, on the order of 10^−10^ per site per year, are still lower than the 4.66 × 10^−9^ site^−1^ year^−1^ core-genome rate estimated by Reno et al. for the archaeon *Sulfolobus islandicus* [126]. The two estimates are not directly equivalent because the former represents synonymous-site divergence in a single gene whereas the latter represents genome-wide nucleotide substitution. Nevertheless, synonymous sites are generally less constrained by purifying selection than nonsynonymous coding sites, making the low *radA* values noteworthy.

In summary, whole-genome and protein-coding comparisons do not remove the molecular paradox originally revealed by 16S rRNA; in the 2-9-3/*V. marismortui* and NMX12-1/JI20-1 comparisons, they sharpen it.

## 7. Possible Explanations

### 7.1. Contamination by Modern Organisms

Early studies were more vulnerable to contamination than later investigations because many relied on bulk brine, efflorescence, or whole-crystal dissolution and used surface treatments that would not meet current standards. Some early isolates may therefore have represented modern halophiles introduced during sampling or processing. Ethanol immersion and brief flaming, although useful decontamination measures, may not reliably eliminate all surface microorganisms. Contamination remains a plausible explanation for some reports, but it becomes less satisfactory for studies using deeply buried material, rigorous surface sterilization, petrographic screening, and direct extraction of primary fluid inclusions.

### 7.2. Recrystallization and Groundwater Infiltration

Halite formations can undergo ductile deformation under pressure, accompanied by solution-precipitation, folding, diapirism, and recrystallization [77,127,128]. Naturally deformed halite also exhibits fluid-assisted grain-boundary migration and subgrain-rotation recrystallization, with arrays of fluid inclusions observed along some grain boundaries [128]. These processes provide potential pathways for fluid redistribution at microscopic scales even in the absence of obvious macroscopic fractures. During such alteration, fluid inclusions may migrate, leak, or be redistributed, and new inclusions can form during the growth of recrystallized halite. Such inclusions may be primary with respect to the newly grown crystal without being syn-depositional with the original salt bed. Stable-isotope studies provided evidence consistent with the mixing of evaporite brine and meteoric groundwater in some salt deposits [127,129]. The contribution of meteoric water generally decreases with depth in the studied systems. Selecting deeply buried crystals that retain primary growth features, such as chevrons, and then targeting demonstrably primary inclusions greatly reduces this concern and has become an important control in paleomicrobiological studies. Nevertheless, petrographic identification of primary growth features cannot by itself establish that microorganisms presently enclosed within an inclusion have remained isolated since the original depositional event. Recrystallization, microscopic fluid migration, and groundwater infiltration therefore remain possible explanations in particular deposits but are less satisfactory where primary depositional textures and inclusion chemistry are well preserved.

### 7.3. Unknown Survival Mechanisms

Although bacterial endospores have been extensively studied, much less is known about the mechanisms underlying long-term survival of non-spore-forming microorganisms, including the dormancy-like microspheres formed by haloarchaea. Extremophiles repeatedly reveal physiological adaptations that expand known limits of life, so additional survival mechanisms cannot be excluded. One possible mode of long-term survival is that cells enter extremely low-activity states punctuated by rare episodes of repair or maintenance that counter the accumulation of molecular damage. Such episodic metabolism could, in principle, reduce the energetic cost relative to continuous maintenance while permitting molecular repair, but direct evidence for the existence of such a strategy and its ability to sustain viability over geological timescales is lacking.

Episodic metabolism could be examined experimentally using laboratory-generated haloarchaeal microspheres. Microspheres can be rapidly induced by reduced water activity and subsequently maintained in high-salt, nutrient-limited media for prolonged periods [94]. Such populations could be maintained under different temperatures and radiation conditions and sampled longitudinally for ATP content, incorporation of isotopically labeled substrates, activity or expression of repair pathways, DNA and protein damage, and recovery of culturability. Continuous low-level maintenance would predict relatively steady metabolic activity, whereas the episodic-metabolism hypothesis predicts intervals of little or undetectable activity interrupted by transient increases in metabolism and macromolecular repair without sustained growth. Long-term experiments of this type could therefore determine whether microspheres periodically repair accumulated molecular damage and whether such activity measurably extends cellular viability.

### 7.4. Genomic Divergence Underestimated by 16S rRNA

Most molecular comparisons between putatively ancient and modern microorganisms have emphasized 16S rRNA genes because they are highly conserved. DNA–DNA hybridization provides evidence that this marker can understate broader genomic differentiation. Legat et al. [130] reported only 63.6% DNA–DNA relatedness between the Permian isolate *Hcc. salifodinae* BIpT and *Hcc. saccharolyticus* DSM 5350T despite 98.9% 16S rRNA gene identity. This hybridization value is a measure of genomic relatedness rather than direct nucleotide-sequence identity. Park et al. [26] reported a 55-nt insertion in 16S rRNA gene sequences recovered from Cretaceous and Silurian halite that was absent from their Oligocene sample, and therefore not included in aligned comparisons. These observations caution against equating high 16S rRNA similarity with genome-wide identity. As additional ancient isolates are subjected to whole-genome sequencing, the extent of genomic divergence from modern relatives may prove greater than is apparent from 16S rRNA comparisons alone.

## 8. Conclusions

The evidence reviewed here leaves a genuine two-sided problem. Modern contamination is a credible explanation for some early reports, but it becomes less persuasive for studies using deeply buried material, rigorous surface sterilization, petrographic selection of primary crystals, and direct sampling of fluid inclusions. At the same time, no established mechanism readily explains the preservation of viable cells for tens to hundreds of millions of years in a small, nutrient-limited inclusion while spontaneous molecular damage continues to accumulate.

Resolving the paradox requires experiments that constrain both geological history and biological history in the same specimen. Future studies should combine documented primary halite and inclusion petrography with modern contamination controls, direct in situ imaging and chemistry, cultivation where possible, and whole-genome analysis of recovered organisms. The broader significance is the boundary condition these claims place on cellular longevity: a viable cell must retain enough molecular information and repair capacity to restart life after prolonged metabolic restriction. Whether ancient halite ultimately reveals extraordinary persistence or an unrecognized route of later entry, it remains a stringent natural test of our understanding of dormancy, molecular maintenance, and the limits of cellular survival.

## Figures and Tables

**Table 1:** Chronological list of reports of microbiological isolations from ancient halite.

Site	Age	Organisms	Surface Sterilization and Culture Methods	Reference
Carey Salt Mine, Hutchinson, KS	Permian 277–283 Myr	Gram-positive halophilic diplococcus	70% alcohol for 15 s	[13]
Irkutsk, NY	Precambrian 650 Myr	2 unidentified species	Large crystal in Bunsen burner flame for 45 s. Drop into liquid media to dissolve till broth was saturated with NaCl. Tested with Pyocyaneus (*Pseudomonas* *aeruginosa*)	[14]
Meyers, NY	Silurian	3 unidentified species	Large crystal in Bunsen burner flame for 45 s. Drop into liquid media to dissolve till broth was saturated with NaCl. Tested with Pyocyaneus (*Pseudomonas aeruginosa*)	[14]
Saskatchewan, Canada	Devonian	6 unidentified species	Large crystal in Bunsen burner flame for 45 s. Drop into liquid media to dissolve till broth was saturated with NaCl. Tested with Pyocyaneus (*Pseudomonas aeruginosa*)	[14]
Zechstein halite, Germany	Permian 252.5–258 * Myr	“*Bacillus circulans*” “*Pseudomonas halocrenaea*”	Large crystal in Bunsen burner flame for 45 s. Drop into liquid media to dissolve till broth was saturated with NaCl. Tested with Pyocyaneus (*Pseudomonas aeruginosa*)	[14,15]
Zechstein halite, Germany	Permian 252.5–258 Myr	Mostly Bacillaceae members, other rods, Gram-positive diplococci	1 mg/L Cl2 for 30 min, 80% ethanol for 30 min, or same as Dombrowski	[16]
Winsford salt mine, Cheshire, England and Boulby potash mine, Cleveland, England	Triassic Period, 195–225 Myr; Permian Period, 252.5–258 Myr	*Hbt. saccharovorum Hbt. salinarum* *Haloarcula* *Halococcus*	Newly exposed crystals submerged in absolute ethanol for 4–5 h and dissolved in media, but other samples were untreated.	[17]
Bad Ischl salt mine, Austria	Permian Period, 245–280 Myr	*Hcc. Salifodinae* *Halobacterium*	Dipping in ethanol and flaming; dissolved in media	[18,19]
Salado Formation, New Mexico	Permian Period, 250 Myr	*Virgibacillus*	10 M NaOH, 10 M HCl, 5 min each; inclusion extracted with syringe	[1]
Bad Ischl-Perneck, Austria; Altaussee, Austria; Berchtesgaden, Germany	Permo-Triassic Period, 245–280 Myr	*Hcc. salifodinae*	Flaming with a Bunsen burner and monitoring temperature with an infrared thermometer; crystals dissolved	[20]
Bad Ischl salt mine, Austria	Permian Period, 245–280 Myr	*Hcc. dombrowskii*	Flaming with a Bunsen burner and monitoring temperature with an infrared thermometer; crystals dissolved	[21]
Death Valley, California	Pleistocene Epoch, 97 Kyr	*Hbt. salinarum*	Inclusions were extracted using aseptic microdrill/micropipette after ethanol treatment	[22]
Altaussee, Austria	Permian Period, 245–280 Myr	*Hbt. noricense*	Flaming with a Bunsen burner and monitoring temperature with an infrared thermometer; crystals dissolved	[23]
Sergipe Basin, Brazil	Cretaceous Period, 121–122 Myr	*Halobacterium* *Natronobacterium*	10 M NaOH, 10 M HCl, 5 min each; crystals dissolved in media	[24]
Death Valley, California	Pleistocene Epoch, 22–34 Kyr	*Halorubrum, Natronomonas, Haloterrigena*	10 M NaOH for 5 min each; crystals dissolved in media	[25]
Salar Grande, Chile	Pliocene Epoch, 1.8-5.3 Myr	*Hbt. noricense*	6% (*w*/*v*) sodium hypochlorite, 10 M NaOH, 10.53 M HCl, 70% (*v*/*v*) ethanol, 20 min each; crystals dissolved in media	[26]
Penobsquis Mines, New Brunswick, Canada	Mississippian, 350 Myr	Multiple anaerobic curved rods/vibrio	Rinsing in 70% alcohol, drying in UV hood for 10 min, extracting center portions, crushed and dissolved in anaerobic medium	[27]
Yungying salt mine, China	Eocene Epoch, 38–41 Myr	*Halobacterium, Halolamina*	10 M NaOH, 10 M HCl, 5 min each; center of crystals chiseled out and dissolved	[28]
Yunying Depression in Jianghan Basin of central China	Eocene Epoch, 33.9–48.6 Myr	*Oceanobacillus*	10 M NaOH, 10 M HCl, 10 min each; crushed with mortar and pestle	[29]
Qianjiang Depression, China	Cretaceous Period, 123 Myr	*Hbt. hubeiense*	10 M NaOH, 10 M HCl, 5 min each; center of crystals chiseled out and dissolved	[4]

* Note: Ages of Zechstein samples have been standardized according to current stratigraphic assignments [30]. Numerical ages reported in older microbiological studies may differ because of revisions to the geological timescale. Ages of the Austrian and German Haselgebirge samples are taken from [20].

**Table 2:** Quantitative estimates relevant to microbial survival over geological timescales.

Process or Observation	System	Reported or Calculated Timescale	Type of Estimate	Interpretive Limitation
^40^K radiation model [58]	Halophilic bacterial spores in a modeled halite fluid inclusion	17–34 Myr at 7 g/L K; 55–109 Myr at 2.2 g/L K	Measured radiation-inactivation constants + dose model	Assumes no active repair during dormancy, a 10-µL inclusion, 4000 CFU/mL initially, radiation is only one damage process.
Permafrost DNA and respiration [64]	Environmental bacterial communities in frozen sediments	4 kb DNA and respiration detected at 400–600 kyr; neither detected at 740 kyr	Environmental molecular and respiration observations	Supports persistence to ~0.5 Myr in the studied permafrost; does not define a universal bacterial or spore survival limit.
Peptide-bond hydrolysis [67]	Amino acids in deep-sea sediments	~1–2 Myr hydrolysis half-life in calcareous sediments	Geochemical molecular-diagenesis estimate	Not a microbial viability measurement; hydrolysis may be slower in non-carbonate sediments and depends on matrix conditions.
Aspartate racemization [12]	*G. stearothermophilus* spores	Half-life of 40–421 yr at 0–15 °C; ~220 yr at 4 °C	High-temperature experiment + kinetic extrapolation	Predicted 50% mortality times; dependent on extrapolation and environmental chemistry.
Thermal-inactivation extrapolation [11]	Thermophilic *Bacillus*-group spores	Extrapolated D-values ~1.9 × 10^9^–1.9 × 10^12^ yr at 25–40 °C	High-temperature inactivation kinetics extrapolated downward	A cautionary counterexample: the author called the upper values nonsensical and noted that brine-specific data were lacking.
Cultivable spores in sediments [69,70,71]	Thermophilic and heterotrophic endospore populations	Half-life of ~330 yr; 350 yr; calculated ~277–533 yr	Environmental viable/culturable abundance decay	Population-level loss of viability/culturability; burial, germination, deposition, and ecology may contribute.
DPA-derived endospore abundance [72]	Global marine-sediment dataset	Older segment: concentration only halved across ~20 Myr	Segmented regression of DPA-derived abundance vs. sediment age	Not a measured viability half-life; cross-site abundance integrates heterogeneous depositional and thermal histories.
Illustrative halite population calculation (this review)	Hypothetical entrapped spore population	~3.7 × 10^6^ spores/metric ton initially; <1 expected after ~11,600 yr	Author calculation using a 533 yr half-life based on viability decline in lake-floor sediments	Assumes 4000 CFU/mL brine, 10% survival/representation after entrapment, 2% fluid volume, and constant first-order decay.
LTSP extinction modeling [84]	21 heterotrophic bacterial taxa in long-term stationary phase	Modeled population extinction ~1–100,000 yr	Laboratory mortality trajectories + population model	Not a sealed halite inclusion and not direct observation over the extrapolated interval; persistence varies strongly among taxa.
*Dunaliella*-supported energy budget [98]	Miniaturized prokaryote supported by glycerol from one Dunaliella cell	~12 Myr published estimate	Energy-budget calculation	Assumes a 10 µm *Dunaliella* cell, 5.5 M glycerol, a 10-fg-C prokaryote, and a specified repair-metabolism demand; strongly assumption-dependent.

Note: These estimates are not directly interchangeable. Some quantify loss of culturability or population abundance, others quantify molecular damage, radiation exposure, or energy supply, and several depend on extrapolation beyond experimentally observed timescales. The table therefore summarizes quantitative constraints and comparisons rather than a set of equivalent maximum survival ages. Taken together, the estimates show both the multiplicity of processes relevant to long-term persistence and the strong dependence of inferred timescale on mechanism, environment, and model assumptions.

**Table 3:** 16S rRNA identity, OrthoANI, and genome alignment coverage between putatively ancient archaeal genomes and that of a modern relative.

Pair	Nominal Ages	16S Identity	OrthoANI	Aligned Genome Coverage
NMX12- 1–JI20-1	250/123 Ma	100.000%	96.86%	57.1/58.4
NMX12-1–*Hbt. noricense*	250/250 Ma	99.861%	90.14%	43.1/44.7
NMX12-1–Korean	250 Ma/modern	99.792%	88.80%	46.2/49.7
JI20-1–*Hbt. noricense*	123/250 Ma	99.861%	89.90%	45.1/47.8
JI20-1–Korean	123 Ma/modern	99.792%	88.75%	47.7/50.2
*Hbt. noricense*–Korean	250 Ma/modern	99.653%	88.25%	44.5/49.6

Note: Genomic sequences used in the analyses were obtained from NCBI RefSeq: GCF_001488575.1 (*Hbt. hubeiense* JI20-1), GCF_040435975.1 (*Halobacterium* sp. NMX12-1), GCF_021233435.1 (*Hbt. noricense*), and GCF_049705405.1 (Korean isolate). Pairwise genomic similarities were calculated from the corresponding genome assemblies.

**Table 4:** *radA* comparison between putatively ancient archaeal genomes and that of a modern relative.

Pair	AA Identity	*K_s_*	Age Difference	Apparent *r_s_* Based on Age Difference
NMX12 -1–JI20-1	99.417%	0.06642	127 Myr	5.23 × 10^−10^
NMX12-1–*Hbt. noricense*	98.251%	0.17546	0 Myr	N/A
NMX12-1–Korean	97.376%	0.14195	250 Myr	5.68 × 10^−10^
JI20-1–*Hbt. noricense*	97.668%	0.21418	127 Myr	1.69 × 10^−9^
JI20-1–Korean	97.376%	0.16299	123 Myr	1.33 × 10^−9^
*Hbt. noricense*–Korean	95.918%	0.17856	250 Myr	7.14 × 10^−10^

Note: Synonymous substitutions per synonymous site (*K_s_*) were estimated from nucleotide sequence differences at synonymous sites. *r_s_* was calculated by dividing *K_s_* by the difference in geological ages between the compared samples. Values are expressed as substitutions per synonymous site per year.

## Data Availability

The genomic sequences analyzed in this review were obtained from publicly available databases, including the NCBI GenBank and RefSeq repositories (https://www.ncbi.nlm.nih.gov/). The comparative genomic analyses presented in Table 3 and Table 4, including 16S rRNA sequence identity, average nucleotide identity (OrthoANI), genome alignment coverage, and RadA sequence comparisons, were performed using these publicly available sequences. The results of these analyses are provided in Table 3 and Table 4. No new genomic sequences were generated in this study.
